# Update on histone deacetylase inhibitors in peripheral T-cell lymphoma (PTCL)

**DOI:** 10.1186/s13148-023-01531-8

**Published:** 2023-08-02

**Authors:** Guang Lu, Shikai Jin, Suwen Lin, Yuping Gong, Liwen Zhang, Jingwen Yang, Weiwei Mou, Jun Du

**Affiliations:** 1 Department of Hematology, West China Hospital, Sichuan University, Chengdu, 610041 Sichuan People’s Republic of China; 2 Department of Hematology, Shengli Oilfield Central Hospital, Dongying, 257034 Shandong People’s Republic of China; 3 Department of Clinical Medicine, Shanghai Jiao Tong University School of Medicine, Shanghai, 200025 People’s Republic of China; 4 Clinical Research Institute, Shenzhen Peking University – The Hong Kong University of Science and Technology Medical Center, Shenzhen, 518036 Guangdong People’s Republic of China; 5 Department of Pediatrics, Shengli Oilfield Central Hospital, Dongying, 257034 Shandong People’s Republic of China; 6 Department of Hematology, Renji Hospital, School of Medicine, Shanghai Jiao Tong University, Shanghai, 200127 People’s Republic of China

**Keywords:** Histone deacetylase, HDACi, PTCL, Romidepsin, Belinostat, Chidamide

## Abstract

Peripheral T-cell lymphomas (PTCLs) are a group of highly aggressive malignancies with generally poor prognoses, and the first-line chemotherapy of PTCL has limited efficacy. Currently, several novel targeted agents, including histone deacetylase inhibitors (HDACis), have been investigated to improve the therapeutic outcome of PTCLs. Several HDACis, such as romidepsin, belinostat, and chidamide, have demonstrated favorable clinical efficacy and safety in PTCLs. More novel HDACis and new combination therapies are undergoing preclinical or clinical trials. Mutation analysis based on next-generation sequencing may advance our understanding of the correlation between epigenetic mutation profiles and relevant targeted therapies. Multitargeted HDACis and HDACi-based prodrugs hold promising futures and offer further directions for drug design.

## Introduction

Peripheral T-cell lymphomas (PTCLs) are derived from mature T-cells or NK/T-cells and represent a group of non-Hodgkin lymphomas. Mature T-cell and NK cell neoplasms are classified into 30 subtypes, and among them, peripheral T-cell lymphoma not otherwise specified (PTCL-NOS), nodal T-follicular helper cell lymphoma (T-FHCL), and anaplastic large cell lymphoma (ALCL) are common subtypes of PTCL [1].

The CHOP (cyclophosphamide, doxorubicin, vincristine, and prednisone) regimen is the first-line chemotherapy for PTCLs. However, CHOP regimen research was previously based on practice with aggressive B-cell lymphomas [2]. Although CHOP regimen has a remission rate of 50%–65% in PTCL, brief response and high risk of relapse are the two primary problems [3]. In one of the largest international prospective T-cell projects, out of 937 PTCL patients who received an active first-line treatment, 633 (68%) were identified as refractory or relapsed patients with a 3-year overall survival (OS) rate not reaching 30% [3]. High-dose chemotherapy and hematopoietic stem cell transplantation can improve prognosis as consolidation in first or second remission, yet strictly limited to patients with tolerable body status and chemotherapy sensitivity [3]. As a result, for patients with PTCL, the CHOP regimen exhibits an unmet need for effective treatment. No optimal standard of treatment for PTCLs has been widely accepted, so effective novel therapies are urgently warranted. With further insights into molecular pathogenesis, targeted therapy becomes an alternative therapeutic option for PTCLs.

**Fig. 1:**
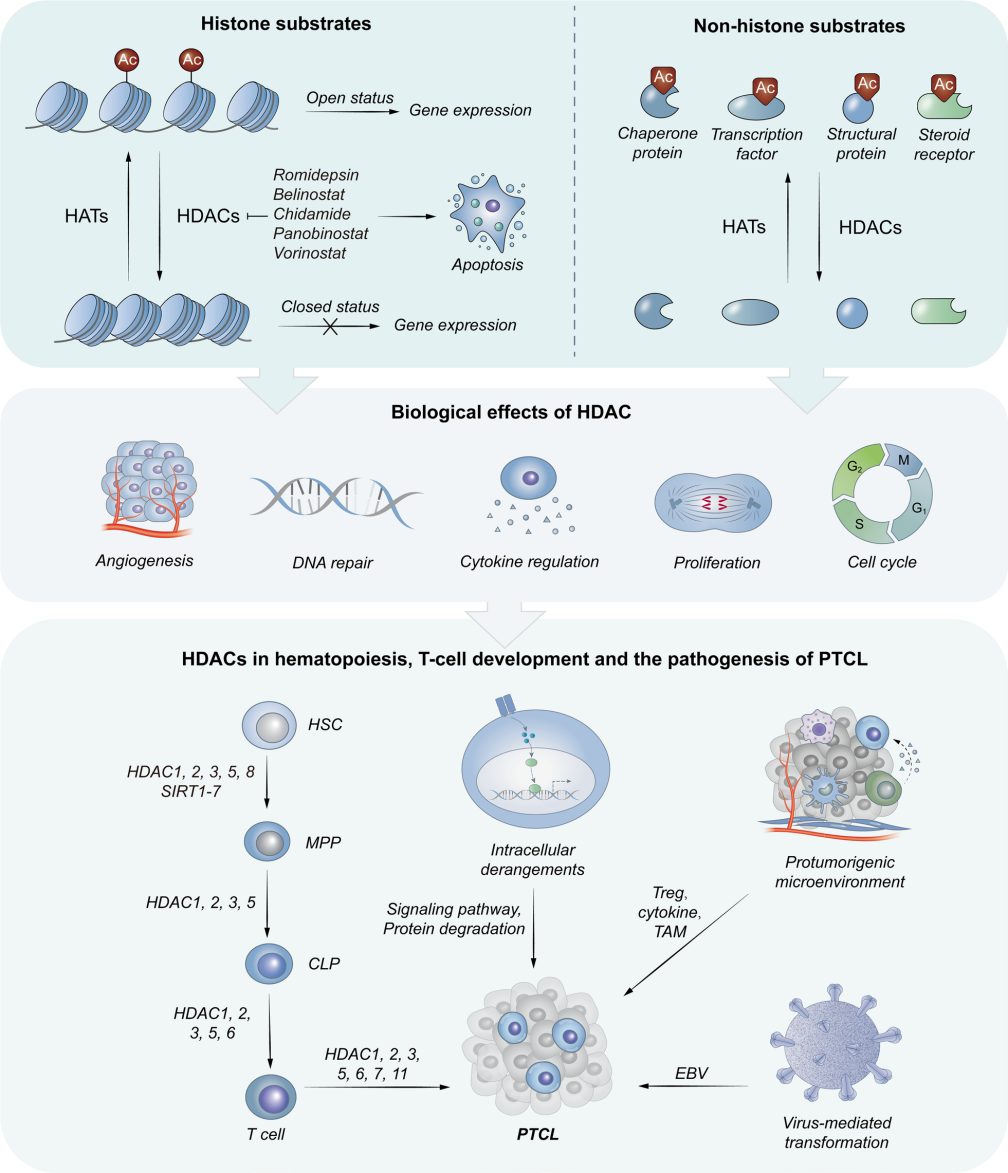
The histone or non-histone substrates for and the integrated biological effects of HDACs. The acetylation of histone substrates modulates the chromatin structure to reduce the accessibility to transcriptional regulatory proteins and subsequent gene expression. For non-histone substrates, HDACs have an impact on their activity by acetylating. In general, HDACs contribute to proliferative effects. Moreover, HDACs play a pivotal role in hematopoiesis and T-cell development. Abnormal expression or activity of HDACs is involved in the malignant transformation of PTCL in multiple ways, including intracellular derangements, protumorigenic microenvironment, and virus-mediated transformation

Due to the aberrant activity and expression of histone deacetylases (HDACs) in tumor cell growth, HDACs are widely studied as therapeutic targets for PTCLs. HDACs mainly function in transcription regulation by removing acetyl groups from the ε-amino groups of the lysine residues of histone tails [4, 5]. Reduced positivity of histone tails contributes to chromatin condensation, hindering the accessibility of transcription factors to DNA. As transcriptional corepressors, HDACs induce a closed status of the nucleosome, resulting in gene suppression. Besides histone substrates, HDACs also regulate the stability and activity of non-histones via post-translational deacetylation, as shown in Fig. 1. Chaperone proteins, transcription factors, structural proteins, and steroid receptors are the principally non-histone substrates of HDACs, including nuclear factor κB (NF-κB), p53, GATA1, GATA2, STAT3, and heat shock protein 90 (Hsp90) [4, 5]. Non-histone protein deacetylation plays a vital role in physiology and pathogenic cellular processes, such as gene transcription, signal transduction, protein folding, autophagy, DNA repair, cell proliferation, and metabolism [6]. Histone acetyltransferases (HATs) reverse these processes by adding acetyl groups [7, 8]. However, HATs and their inhibitors are paid less attention because of their inadequate medical value for PTCLs.

Regarding mammals, 18 kinds of HDACs have been subdivided into four classes based on their homology to yeast HDACs—sequence motifs, cellular location, tissue specificity, and enzymatic activity, as summarized in Table 1. In Class I, HDAC1, 2, and 3 are ubiquitously distributed in human tissues, while HDAC8 is located in smooth muscles and affects their contractility [9]. Of note, PD-L1 nuclear translocation has been reported as being HDAC2-dependent, providing a hint for research to identify targeted agents [10]. HDAC3 uniquely localizes to the mitotic spindle during mitotic progression to maintain proper kinetochore-microtubule attachment and chromosome alignment [11]. In addition, HDAC3 is essential for inflammatory response during host defense against bacterial infection by facilitating TNFα-mediated NF-κB activation [12]. Furthermore, HDAC8 has been found to have an anti-apoptotic effect via repressing transcription of the proapoptotic protein Bcl-2-modifying factor [13]. Due to different sequence homology and domain organization, Class II HDACs were further subdivided into IIa and IIb subclasses [5]. Subclass IIa (HDAC4, 5, 7, and 9) is characteristic of signal-dependent nucleocytoplasmic shuttling and tissue-specific expression [5, 14]. Subclass IIb (HDAC6 and 10) has two catalytic HDAC domains and is distributed in limited tissue types with a cytoplasmic localization [5]. HDAC6 is well known for its deacetylating function on specific cytosolic non-histone substrates that participate in the tumorous genesis, development, and metastasis. The common substrate types are α-tubulin, cortactin, peroxiredoxin, Hsp90, and heat shock transcription factor-1 (HSF-1) [15]. Unlike HDAC6 as an acetyllysine deacetylase, HDAC10 is an N8-acetylspermidine deacetylase, which is associated with dysregulated polyamine metabolism and relevant neoplastic diseases, such as colon cancer, prostate cancer and neuroblastoma [16]. Several studies demonstrated that HDAC10 promotes cell survival through autophagy in response to chemotherapeutic drugs and pathogen infection [16, 17]. Hence, suppression of HDAC10 autophagy may be a novel strategy to sustain the cytotoxicity of cancer chemotherapy, especially for the treatment of advanced-stage neuroblastoma [16]. HDAC11 is the only class IV HDAC due to a unique sequence motif [5, 18]. HDAC11 has deacetylase activity and more efficient defatty-acylase activity, which is significant for lipid metabolism [18]. It is promising for HDAC11 to be an emerging therapeutic target for chronic metabolic diseases [19]. A pan-cancer analysis found that HDAC11 is not a pure oncogenic factor but plays a protective prognostic role in specific cancers (e.g., kidney renal clear cell carcinoma and rectum adenocarcinoma) [20]. The sirtuins (SIRT1-7) comprise the Class III HDACs, taking NAD^2+^ rather than Zn^2+^ as its cofactor to facilitate deacetylase activity [5, 21–24]. In particular, SIRT3-5 is located in the mitochondrial matrix and is associated with metabolic control [22]. Through affecting reactive oxygen species (ROS) and even reactive nitrogen species (RNS), SIRTs regulate the metabolism, cellular reproduction, aging, autophagy, and mitophagy, which are considered promising candidate targets for the exploitation of antitumoral therapies [22–24].

**Table 1:** Classification and features of HDACs

Classification	Subtypes	Tissue specificity	Cellular location	Function	References
Class I	HDAC1, 2	NA	Nucleus	Mediate DNA damage response, regulate cardiac morphogenesis, growth, and contractility, and repress cytokine production	[7, 14]
	HDAC3	NA	Nucleus, cytoplasm, and plasma membrane	Mediate DNA repair, endochondral bone formation, mitosis, and inflammatory response	[4, 7, 11, 12, 14]
	HDAC8	Smooth muscles	Nucleus and cytoplasm	Affect contractility in smooth muscles and mediate anti-apoptosis	[7, 9, 13, 14]
Class II	IIa (HDAC4, 5, 7, 9)	Brain, heart, lungs, placenta, pancreas, skeletal muscles, and thymus	Nucleocytoplasmic shuttling	Regulate myocyte, osteocyte, and cardiomyocyte differentiation	[5, 7]
	IIb (HDAC6, 10)	Heart, skeletal, muscles, brain, liver, spleen, and kidney	Cytoplasm	Regulate angiogenesis, cell motility, adhesion, polyamine metabolism, and antiviral innate immune response	[5, 7, 16, 17]
Class III	Sirtuins (SIRT1-7)	NA	Nucleus and cytoplasm, mitochondrion	Regulate mitochondrial function, autophagy and mitophagy, and mediate DNA repair	[5, 7, 23]
Class IV	HDAC11	NA	Nucleus	Regulate lipid metabolism and immunoregulation	[5, 18]

*NA* Not applicable, *HDAC* Histone deacetylase, *SIRT* Sirtuins

## HDACs in hematopoiesis, T-cell development, and the pathogenesis of PTCL

HDACs involve in hematopoietic multilineage development. In Class I, HDAC1 and 2 are regarded as necessary for hematopoietic stem cell (HSC) formation, survival, and homeostasis [25]. In addition, HDAC1 exhibits dynamic expression changes and is involved in lymphoid lineage commitment determination during hematopoiesis and cell differentiation [25]. HDAC1 and 2 repress the Runx3-CBFβ complexes that induce CD8 lineage programs in CD4^+^ T-cells, leading to the maintenance of CD4 lineage integrity. Loss of either HDAC1 or HDAC2 alone has little effect, but dual inactivation at the early stage results in developmental arrest and reduction in thymocyte numbers and peripheral T-cells, without response to TCR signaling [25–27]. HDAC3 is a negative regulator in normal human HSC expansion and development. During T-cell development, HDAC3 is involved in multiple biological processes, such as CD4 and CD8 lineage commitment, positive selection, and peripheral T-cell maturation [25]. Knockdown of HDAC3 in mice blocked hematopoietic progenitor differentiation toward lymphoid lineages and hinder DNA replication [25, 28]. HDAC3-deficient Peripheral T-cells without HDAC3 have an intrinsic defect in their ability to produce TNF efficiently after TCR/CD28 stimulation [25]. HDAC8, upregulated in long-term HSCs and multipotent progenitor cells, is essential for the quiescence, maintenance, and functional integrity of long-term HSCs by modulating p53 activity [25, 29, 30]. In Class II, HDAC5 was reported as a negative regulator for HSC homing and engraftment via regulating p65 deacetylation and CXCR4 surface expression [31]. Besides, Class II HDACs play a pivotal role in the regulation of TCR-mediated apoptosis during T-cell negative selection through interaction with the transcript factor MEF2D [8]. HDAC7 deletion triggers a truncated repertoire of TCR Jα segments, promotes thymocyte apoptosis, and causes inefficient positive selection [25, 32]. In Class III, SIRT1-7 facilitate the maintenance of HSC homeostasis and protect HSCs against aging through different signaling pathways [25]. In Class IV, antagonistic to HDAC6, HDAC11 serves as a transcription repressor of IL-10 expression in antigen-presenting cells, which is essential for T-cell activation [33]. A murine model revealed that activation of resting T-cells triggered lower expression of HDAC11, which enhanced proliferation, proinflammatory cytokine production, and effector molecule expression [34].

HDACs also play a significant role in the malignant transformation of hematologic diseases [25]. Abnormal expression or activity of HDACs has been found in several hematologic malignancies, such as diffuse large B-cell lymphoma (DLBCL), follicular lymphoma (FL), and chronic lymphocytic leukemia (CLL), contributing to the epigenetic silencing of tumor suppressor genes and oncogene activation [8, 35]. In a murine model, HDAC1 or 2 haploinsufficiency triggers T-cell lymphomas with global histone acetylation and chromosomal instability [25, 27]. Low levels of HDAC7 and HDAC1 or 2 activity are essential for T-cell lymphoma development [35]. High expression of HDAC1, 2, and 6 frequently occurs in cases with PTCL [36]. Moreover, HDAC6 overexpression in PTCL is associated with poor outcomes [35]. The intrinsic alters of HDACs based on molecular subtypes of PTCL are temporarily under-reported. The pathogenesis of PTCL is summarized as the following three aspects: (i) intracellular derangements, (ii) protumorigenic microenvironment, and (iii) virus-mediated transformation [37]. The involvement of HDACs in the pathogenesis of PTCL from the above aspects is discussed as follows.

### Intracellular derangements

HDACs are extensively involved in the regulation of downstream gene networks and signaling pathways, mainly relying on the deacetylation of non-histone substrates such as transcription factors and signaling mediators. For instance, HDAC1, 2, and 3 inhibit transcription of STAT3 target genes in the JAK/STAT pathway, contributing to epigenetic tumor suppressor gene silencing and decreased induction of cell growth arrest or apoptosis [5, 35]. In addition, HDAC1 induces the deacetylation of p53 to repress its function and reduce the level of apoptosis signaling [5]. The acetylation of the K382 site induced by HDACi leads to the decreased degradation-promoting activity of mouse double minute 2 homolog (MDM2) and increased p53 stabilization [38]. In terms of p53 mutants, HDAC1 and 2 are still able to integrate their expression, which was reported in murine pancreatic cancer cells [39]. Moreover, HDAC3 participates in the NF-κB activation via mediating TNF expression in the TCR/CD3 pathway [12, 25, 37].

HDACs promote protein degradation, due to direct competition between acetylation and ubiquitylation for modification of the same lysine residues [6]. TET2 gene mutation frequently occurs in the early stage of the pathogenesis of PTCL. HDAC1 and 2 mediate the deacetylation of the TET2 protein and cause its degradation via the ubiquitin–proteasome pathway [40]. Interestingly, TET2 proteins in breast cancer cells can recruit HDACs to the PD-L1 gene promoter to suppress its transcription, which may be independent of DNA demethylation [41].

### Protumorigenic microenvironment

Regulatory T-cells (Tregs) can be selectively recruited by PTCL to dampen the antitumor immune response and promote tumor survival [37]. Different categories of Tregs have been recorded in PTCL subtypes. Suppressor or malignant Tregs can be found in PTCL-NOS and ALCL. Incompetent Tregs, typically with a resting phenotype, emerge into AITL [37]. FOXP3, as a vital transcription factor of Treg, is regulated by various post-translational modifications, including lysine acetylation induced by HDACs [42]. HDAC5 plays a pivotal role in Treg homeostasis [43]. Tregs bereft of HDAC3 or 5 display reduced suppressive function and decreased FOXP3 [25, 44]. Deficiencies in HDAC3 or HDAC5 impair the ability of conventional T-cells to convert into induced Tregs [25, 44]. As opposed to HDAC3 and 5, HDAC6, 9, 10, or 11-defect Tregs exhibit enhanced immunosuppressive capacity and higher expression or acetylation of FOXP3 [20, 42, 43]. Furthermore, HDAC11-defect T-cells are less susceptible to suppression by Tregs in vitro [34]. A meta-analysis based on subgroups of lymphomas demonstrated that there was a significant association between high Tregs and longer OS in Hodgkin lymphoma (HL), DLBCL, and NK/TCL, but it was not discovered in TCL or FL [45].

Cytokine milieu, as one of the critical contributors to lymphomagenesis, plays a pivotal role in defining the T-cell phenotype, modulating gene expression, and integrating T-cell plasticity [37, 46]. Deletion of HDAC1 in Th1 cells and CD8^+^ T-cells showed increased production of IFN-γ [43]. HDAC2 and 3 are recruited by Kruppel-like factor 4 (KLF-4) at the vascular endothelial growth factor (VEGF) promoter to participate in transcriptional repression of VEGF, which has been first reported in breast cancer [47]. Loss of KLF-4 or HDAC2 and 3 may contribute to the angiogenesis of the development of tumors [47]. DAC3-deficient conventional T-cells produced large amounts of IL-2, IL-6, and IL-17 [44]. Deficiency in HDAC5 caused reduced IFN-γ in CD8^+^ T-cells but did not affect the proliferation or cytokine expression of CD4^+^ T-cells [25, 43]. CD4^+^ T-cells lacking HDAC7 produced elevated levels of IL-2 and IFN-γ [43]. Analogously, T-cells missing HDAC11 promoted cell proliferation and proinflammatory cytokine production (e.g., IL-2 and IFN-γ) and suppressed lymphomatous progression in the murine model [25]. The correlation between serum cytokines and the prognosis of PTCL subtypes was explored by a study, which measured 34 cytokines in serum samples from 121 PTCL patients (PTCL-NOS, *n* = 55; AITL, *n* = 44; ALK^−^ ALCL, *n* = 22) [48]. In the AITL group, only IL-10 showed the prognostic value and has been suggested to promote lymphomagenesis through dysregulation of monocyte differentiation, aberrant activation of the JAK2 pathway, and down-regulation of antigen presentation inducing immune escape [48]. In the ALK^−^ ALCL group, higher expression of IFN-γ, IL-8, IL-10, IL-17, IL-23, IP-10, MCP-1, MIP-1β, and RANTES were associated with shorter OS, while IFN-γ, IL-8, and RANTES had a further association with a lower CR rate, which indicated that the recruitment of tumor-associated macrophages (TAMs) may be involved in the lymphomagenesis [48]. Cytokines including IFN-γ, IL-7, and IL-23 are associated with poor prognosis in the PTCL-NOS group [48].

Tumor-associated macrophages (TAMs) are regarded as contributors to the poor prognosis of malignancy and are often associated with immune escape and early local or metastatic relapse [49]. One of the VAV1 fusion oncogenes, Vav1-Myo1f was found functioning in inducing recruitment and enrichment of TAMs to the protumorigenic microenvironment in PTCL [50]. Another study revealed that in melanoma, breast cancer, and lung cancer, low-dose pan-HDACi TSA may abrogate the functions of pro-tumoral TAMs to facilitate tumor regression both in vitro and in vivo, with decreased M2-like macrophages and increased M1-like macrophages, which can be a clue for the therapeutic intervention of PTCL [49].

### Virus-mediated transformation

Epstein-Barr Virus (EBV) is a γ-herpesvirus and is regarded as an etiological factor in the tumor progression of multiple human malignancies, including lymphoma, gastric carcinoma, and nasopharyngeal carcinoma (NPC) [51]. EBV was reported a frequency of 30–100% in PTCL [52]. Except for occasional PTCL-NOS, EBV infects bystander B cells rather than the neoplastic T lymphocytes in PTCL, which means that EBV plays an indirect role in the pathogenesis of PTCL [37]. A study showed that only AITL was strongly associated with EBV and assigned a score of 3 (defined as “with positive large CD30^+^ B-cells”) among all subtypes of T-cell lymphoma enrolled [53]. Besides, EBER (EBV-encoded RNA) positivity in the T-cell lymphoma group was statistically and significantly associated with relapse (*p* < 0·01) [53]. However, up to now, how EBV induces the occurrence or development of AITL has remained unclear and the role of involved HDACs also has not been fully understood [51, 53]. Notably, the HDAC-related EBV infection of other cell lines, such as Burkitt lymphoma [54] and NPC [55], has been reported. In Burkitt lymphoma, Class I HDACi romidepsin potently induces EBV lytic cycle and mediates enhanced cell death with ganciclovir through inhibiting HDAC1, 2, and 3 and upregulating p21 [56]. In the NPC cell lines, EBV latent protein LMP1 upregulates STAT5A and recruits HDAC1/2 to the locus of the CEBPA gene, which is involved in neoplastic plasticity regulation and cellular dedifferentiation [55]. In addition, HDACis (romidepsin and chidamide) have been confirmed their effects in restoring CEBPA expression and reversing cellular dedifferentiation in vitro in EBV^+^ NPC [55]. Human T-cell leukemia virus type 1 and Kaposi’s sarcoma-associated herpesvirus were reported in isolated PTCL cases [51]. However, the relationship between HDACs and oncogenic viruses excluding EBV has remained a mystery in PTCL.

## Histone deacetylase inhibitors

**Fig. 2:**
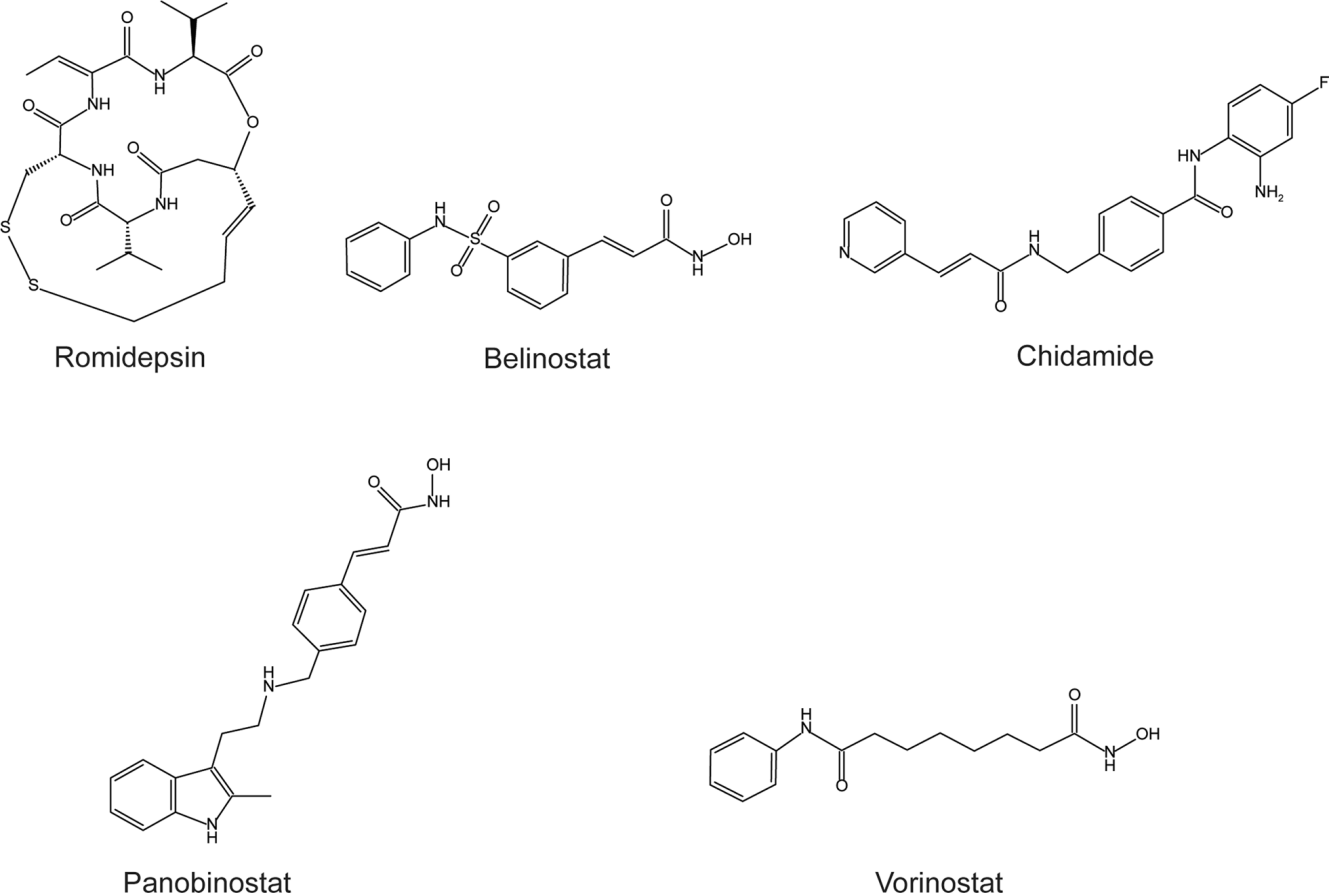
Five approved HDACis. To date, five HDACis have been approved by the FDA for the treatment of various cancers. However, only three approved HDACis, including romidepsin, belinostat, and chidamide, are in clinical application for PTCLs

Deregulation of HDACs plays a pivotal role in oncogenesis, and HDACis exert an antitumor effect by facilitating apoptosis, autophagy, and other cell damage processes [21]. HDACis have been confirmed as a class of available targeted agents, used alone or in combination, for various cancers, such as hematological, breast, colorectal, pancreatic, gastric, liver, bladder, and lung cancers, as well as malignant melanoma [15]. There are totally 5 approved HDACis by FDA, but only three are approved agents (romidepsin, belinostat, and chidamide) in clinical application for PTCLs. More details on correlated trials can be found in Table 2 and Fig. 2.

**Table 2:** Clinical trials for HDACis in PTCLs

Agent	Target	Year	Disease	Phase	*N*	Clinical response	Main grade 3/4 AEs	Clinical trial registration number	References
Romidepsin	HDAC1,2	2017	R/R PTCL	II	40	ORR (43%), CRR (25%), mPFS (5.6 months), and mDOR (11.1 months)	Lymphopenia (74%), Neutropenia (54%), Leukopenia (46%), and Thrombocytopenia (38%)	NCT01456039	[59]
Romidepsin + CHOP	HDAC1,2	2022	Untreated PTCL	III	211	ORR (63%), CRR (41%), mPFS (12.0 months), and mOS (51.8 months)	Thrombocytopenia (50%), Neutropenia (49%), Anemia (47%), and Leukopenia (32%)	NCT01796002	[61]
Romidepsin + pembrolizumab	HDAC1,2 and PD-1	2020	R/R PTCL, MF(N = 3)	II	14	ORR (50.0%) and CRR (35.7%)	NA	NCT03278782	[62]
Romidepsin + 5-azacitidine	HDAC1,2 and DNMT	2021	R/R PTCL	II	25	ORR (61%), CRR (48%), mPFS (8.0 months), and mDOR (20.3 months)	Thrombocytopenia (48%), Neutropenia (40%), Lymphopenia (32%), and Anemia (16%)	NCT01998035	[64]
Romidepsin + alisertib	HDAC1,2 and AAK	2020	R/R Lymphoma	I	25	ORR (28%), CRR (12%), and mOS (12.0 months)	Thrombocytopenia (40%), Anemia (28%), and Neutropenia (24%)	NCT01897012	[66]
Romidepsin + bendamustine	HDAC1,2	2019	R/R PTCL	NA	7	ORR (42.9%), CRR (28.6%), and mPFS (7 months)	Thrombocytopenia (42.9%), Neutropenia (28.6%), Anemia (14.3%), and Nausea and vomiting (42.9%)	NA	[67]
Romidepsin + duvelisib	HDAC1,2 and PI3K-δ, γ	2018	R/R PTCL (N = 36) and CTCL	I	63	PTCL: ORR (47.2%, 17/36), and CRR (25.0%); CTCL: ORR (37.0%) and no CR	Neutropenia (15.9%) and Stevens-Johnson syndrome (n = 1, grade 5)	NCT02783625	[69]
Romidepsin + tenalisib	HDAC1,2, PI3K-δ, γ, and SIK3	2021	R/R PTCL (N = 12) and CTCL	I/II	27	PTCL: ORR (75%), CRR (50%), and mDOR (5.03 months); CTCL: ORR (53.3%), CRR (6.3%), and mDOR (3.8 months)	Thrombocytopenia (21%), ALT elevation (18%), and Neutropenia (15%)	NCT03770000	[70]
Romidepsin + pralatrexate	HDAC1,2 and DHFR	2018	R/R Lymphoma (PTCL, N = 14)	I	23	ORR (57%), CR (17%), and mPFS (4.4 months)	Thrombocytopenia (28%), Anemia (24%), and Oral mucositis (14%)	NCT01947140	[71]
Romidepsin + lenalidomide	HDAC1,2 and CRBN	2021	R/R Lymphoma (PTCL, N = 15)	I	45	ORR (49%), CRR (18%), mPFS (5.7 months), mOS (24.0 months), and mDOR (15.7 months)	Thrombocytopenia (53%), Lymphopenia (51%), Neutropenia (49%), and Leukopenia (45%)	NCT01755975	[73]
Romidepsin + lenalidomide + carfilzomib	HDAC1,2, CRBN, and proteasome	2021	R/R Lymphoma (PTCL, N = 13)	I	25	ORR (48%), CRR (20%), mPFS (3.4 months), mOS (26.5 months), and mDOR (10.6 months)	Thrombocytopenia (16%) and Neutropenia (14%)	NCT02341014	[73]
Romidepsin + gemcitabine	HDAC1,2	2016	R/R PTCL	II	20	ORR (30%), CRR (15%), 2-year OS (50%), mOS (22 months), 2-year PFS (11.2%), and mPFS (2.5 months)	Thrombocytopenia (60%), Neutropenia (50%), Anemia (20%), and Transaminase increase (15%)	NCT01822886	[75]
Romidepsin + GDP	HDAC1,2	2019	R/R PTCL (N = 10) and DLBCL	I	20	ORR (50%), no CR, 1-year PFS (15%), mPFS (2.3 months), 1-year OS (42%), and mOS (7.16 months)	Thrombocytopenia (55%), Neutropenia (55%), and Anemia (30%)	NCT01846390	[77]
Romidepsin + liposomal doxorubicin	HDAC1,2	2020	R/R PTCL (N = 11) and CTCL	I	21	PTCL: ORR (27%), CRR (27%), mPFS (2.1 months), mOS (17.5 months), mTTR (3.5 months), and mDOR (4.2 months); CTCL: ORR (70%), CRR (10%), mPFS (6.9 months), mOS (not reached), mTTR (2 months), and mDOR (5.1 months)	Thrombocytopenia (17%), Anemia (13%), and Neutropenia (9%)	NCT01902225	[78]
Belinostat	Class I, II, IV HDACs	2015	R/R PTCL	II	120	ORR (25.8%), CRR (10.8%), mDOR (13.6 months), mPFS (1.6 months), and mOS (7.9 months)	Anemia (10.8%), Thrombocytopenia (7%), Dyspnea (6.2%), and Neutropenia (6.2%)	NCT00865969	[83]
Belinostat + CHOP	Class I, II, IV HDACs	2021	Untreated PTCL	I	21	ORR (86%), and CRR (66.7%)	Neutropenia (30%) and Anemia (17%)	NCT01839097	[87]
Chidamide	HDAC1,2,3,10	2015	R/R PTCL	II	79	ORR (28%), CRR (14%), mPFS (2.1 months), and mOS (21.4 months)	Thrombocytopenia (22%), Leukopenia (13%), and Neutropenia (11%)	ChiCTR-TNC-10000811	[91]
Chidamide	HDAC1,2,3,10	2022	R/R ATLL	IIb	23	ORR (30.4%), CRR (4.3%), mPFS (1.7 months), mDOR (9.2 months), mOS (7.9 months)	Thrombocytopenia (39.1%), Neutropenia (39.1%), Leukopenia (30.4%), and Anemia (17.4%)	NCT02955589	[92]
Chidamide + CHOP	HDAC1,2,3,10	2021	Untreated PTCL	Ib	28	ORR (89.3%), CR/CRu rate (57.1%), and mPFS (14.0 months)	Leukopenia (90.0%), Neutropenia (83.3%), Vomiting (13.3%), Thrombocytopenia (10.0%) and Febrile neutropenia (10.0%)	NCT02809573	[93]
Chidamide + CHOEP	HDAC1,2,3,10	2021	Untreated PTCL	II	113	ORR (60.2%), CRR (40.7%), mPFS (10.7 months), and mDOR (9.2 months)	Leucopenia (69.0%), Neutropenia (69.0%), Anemia (37.2%), Thrombocytopenia (31.0%), and Hypokalemia (15.0%)	NCT02987244	[94]
				Ib	15	ORR (46.7%) and CRR (26.7%)	Leucopenia (60.0%), Neutropenia (66.7%), Anemia (26.7%), Thrombocytopenia (33.3%), Fever (13.3%), and Hyponatremia (13.3%)		
Chidamide + PET	HDAC1,2,3,10	2022	Untreated AITL	II	51	ORR (90.2%), CR/CRu rate (54.9%), mPFS (42.6 months), 2-year PFS (66.5%), and 2-year OS (82.2%)	Neutropenia (32.3%), Lymphopenia (5.8%), and Thrombopenia (5.9%)	NCT03273452	[40]
Panobinostat + bortezomib	Class I, II, IV HDACs and proteasome	2015	R/R PTCL	II	23	ORR (43.5%), CRR (21.7%), DOR (5.6 months), mPFS (2.59 months), mOS (9.90 months)	Thrombocytopenia (68%), Neutropenia (40%), and Diarrhea (20%)	NCT00901147	[95]
Vorinostat + CHOP	Class I, II, IV HDACs	2013	Untreated PTCL	II	14	ORR (85.7%), CRR (85.7%), mDOR (29 months), 2-year PFS (79%), and 2-year OS (81%)	Neutropenia (64%), Thrombocytopenia (14%), and Pain (14%)	NCT00787527	[97]
Vorinostat + lenalidomide + dexamethasone	Class I, II, IV HDACs and CRBN	2014	R/R PTCL	I/II	8	ORR (25%), CRR (12.5%), mPFS (2.2 months), and mOS (6.7 months)	Thrombocytopenia (23%), Leukocytopenia (15%), Anemia (8%), and Neutropenia (8%)	NCT00972842	[98]
Vorinostat + alisertib	Class I, II, IV HDACs and AAK	2020	R/R lymphoma (PTCL, N = 2)	I	34	ORR (11.8%) and CRR (5.9%),	Neutropenia (22%), Leukocytopenia (17%), and Anemia (17%),	NCT01567709	[99]

*NA* Not acquired, *R/R* Relapsed/refractory, *PTCL* Peripheral T-cell lymphoma, *AITL* Angioimmunoblastic T-cell lymphoma, *DLBCL* Diffuse large B-cell lymphoma, *CTCL* Cutaneous T-cell lymphoma, *ATLL* Adult T-­cell leukemia/lymphoma, *ORR* Overall response rate, *CRR* Complete response rate, *CR/CRu* Confirmed/unconfirmed complete response, *mPFS* median progression-free survival, *mOS* median overall survival, *mDOR* median duration of response, *mTTR* median time to response, *CHOP* Cyclophosphamide, doxorubicin, vincristine, and prednisone, *GDP* Gemcitabine, dexamethasone, and cisplatin, *CHOEP* Cyclophosphamide, doxorubicin, vincristine, etoposide, and prednisone, *PET* Prednisone, etoposide, and thalidomide, *DNMT* DNA methyltransferase, *AAK* Aurora A kinase, *ALT* Alanine aminotransferase, *SIK3* Salt-inducible kinase 3, *DHFR* Dihydrofolate reductase, *CRBN* Cereblon

### Romidepsin

Romidepsin (FK228), a selective HDAC1 and 2 inhibitor, has been a research hotspot since it was approved by the US Food and Drug Administration (FDA) for the treatment of PTCLs in patients who have received at least one prior therapy [57]. This approval was mainly based on a phase II trial for single-agent romidepsin in relapsed/refractory (R/R) PTCL, with an objective response rate (ORR) of 25% (33/130), a confirmed/unconfirmed complete response (CR/CRu) rate of 15% (19/130), and a 28-month median duration of response (mDOR) for all responders [57, 58]. The most common grade 3/4 adverse events (AEs) were thrombocytopenia (24%), neutropenia (20%), and infections (19%) [58]. In another phase II in Japanese patients with R/R PTCL, a 43% (17/40) ORR, a 25% (10/40) complete response rate (CRR) and a 5.6-month median progression-free survival (mPFS) were responded [59]. Furthermore, the two major PTCL subtypes demonstrated similar ORRs [AITL, 44% (8/18) vs. PTCL-NOS, 41% (7/17)] [59]. The most common treatment-emergent grade 3/4 AEs were lymphopenia (74%) and neutropenia (54%) [59].

Romidepsin induces cell cycle arrest by raising the expression of the p21 tumor-suppressor gene, which is more sensitive to tumor cells [60]. However, romidepsin mediates apoptosis preferentially in terms of cell lines with reduced p21 expression [60]. In addition, romidepsin induces hyperacetylation of the chaperone protein HSP90, thus degrading the oncoproteins that require HSP90, including mutant p53, RAF-1, and BCR-ABL [60]. Moreover, romidepsin has been found to downregulate the expression of angiogenic-stimulating factors and block the migration and adhesion of endothelial cells to inhibit neovascularization and tumor expansion [60].

Romidepsin exhibits single-agent activity in PTCLs, and the efficacy and safety of this compound in various combination regimens have been widely evaluated. Of note, in a randomized phase III study for previously untreated PTCLs, the therapeutic strategy of romidepsin plus CHOP (Ro-CHOP, *n* = 211) did not achieve marked advantages in efficacy compared to CHOP (*n* = 210), with evaluations in mPFS (12.0 vs*.* 10.2 months), median overall survival (mOS) (51.8 vs. 42.9 months), ORR (63% vs. 60%), and CR/CRu rate (41% vs. 37%) [61]. Additionally, Ro-CHOP increased the frequency of grade ≥ 3 treatment-emergent AEs, including thrombocytopenia (50% vs. 10%), neutropenia (49% vs. 33%), anemia (47% vs. 17%), and leukopenia (32% vs*.* 20%) [61]. Combination regimens for other novel agents with romidepsin in PTCLs have also been explored: 1) The multiple mutations in epigenetic modifier genes (e.g., TET2, IDH2, and DNMT3A) and TCR-related genes (e.g., RHOA and FYN) may impair immunogenicity of PTCL and facilitate immune escape [62]. In a phase II trial (NCT03278782) of pembrolizumab (a check-point PD-1 inhibitor) plus romidepsin in R/R TCL, a 50% (7/14) ORR and a 35.7% (5/14) CRR were recorded but no responses were achieved in the 3 patients with mycosis fungoides (MF) [62]. A higher level of PD-L1 was found in CRs than in PR or SD [62]. 2) A strong synergistic interaction between DNA methyltransferase (DNMT) inhibitor and HDACis has been found in TCL models, through the down-regulation of genes involved in protein and lipid biosynthesis and up-regulation of molecules involved in protein kinase cascade and cell cycle arrest [63]. Markedly, combined romidepsin and 5-azacytidine (an oral DNMTi) demonstrated favorable therapeutic outcomes in patients with T-FHCL, a subtype of PTCL especially vulnerable to epigenetic modifiers, exhibiting an ORR of 80% (12/15), a CRR of 60% (9/15), and lower toxicity [64]. 3) Aurora A kinase (AAK) inhibitor alisertib (MLN8237) and romidepsin appear to be highly synergistic in the TCL model through modulation of mitotic proteins and cytokinesis failure, which has an association with HDAC3 function [65]. In a phase I trial (NCT01897012) in R/R BCL and TCL, the combination of alisertib and romidepsin resulted in an ORR of 28% (7/25) and a CRR of 12% (3/25) [66]. Only one case of AITL was enrolled and achieved no response [66]. 4) A cytotoxic drug bendamustine functions in the promotion of p53-dependent apoptosis with DNA breaks and generation of mitochondrial-mediated ROS and apoptosis in a non-p53-dependent way, which is similar to romidepsin [67]. A study in 7 patients with R/R PTCL showed that romidepsin plus bendamustine attained 2 CRs, 1 PR, and an mPFS of 7 months with nausea and vomiting as the most predominant AE [67]. 5) PI3Ki has been found synergistic effects with HDACi in vitro and in vivo in non-Hodgkin lymphoma (NHL), via induction of DNA damage, downregulation of Mcl-1, and upregulation of Bim protein [68]. Duvelisib, a PI3K-δ,γ inhibitor, combined with romidepsin was evaluated in R/R PTCL and cutaneous T-cell lymphoma (CTCL) in a phase I trial (NCT02783625) [69]. For patients with PTCL, this regimen achieved a 47.2% (17/36) ORR and a 25.0% (9/36) CRR with a lower rate of transaminitis than single-agent duvelisib [69]. Of note, there was a grade 5 AE, Stevens-Johnson syndrome, which was considered possibly treatment-related [69]. A phase I/II study was designed to evaluate a new combination regimen of romidepsin plus tenalisib (RP6530, a highly selective PI3K δ/γ and SIK3 inhibitor) in R/R PTCL and CTCL [70]. Twelve evaluable R/R PTCL patients responded with an ORR of 75% (9/12), a CRR of 50 (6/12) and a 5.03-month median duration of response (mDOR) [70]. 6) The novel antifolate dihydrofolate reductase (DHFR) inhibitor pralatrexate was the first drug approved for patients with R/R PTCL in 2009 [71]. The synergistic effect of pralatrexate and romidepsin was attributed to the inhibition of DNA synthesis and repair, which was studied in vitro in a murine model of human TCL [72]. This combination regimen led to an ORR of 71% (10/14) and a CRR of 29% (4/14) in patients with R/R PTCL in a phase I study (NCT01947140) [71]. For the R/R PTCL group, the median DOR, PFS, and OS were 4.29, 4.4, and 12.4 months, respectively [71]. 7) Cereblon (CRBN) inhibitor lenalidomide has been approved by FDA for various hematologic malignancies with antineoplastic effects of repressing angiogenesis and intensifying immune responses [73]. Lenalidomide and romidepsin had a synergistic or additive effect in specific TCL cell lines via various induced ways, such as induction of apoptosis, increased production of reactive oxygen species, and promotion of endoplasmic reticulum stress [74]. In addition, carfilzomib is an irreversible proteasome inhibitor approved for the treatment of multiple myeloma (MM) [73]. In a combined analysis of two phase I studies (NCT01947140 and NCT01755975), two regimens (A: romidepsin and lenalidomide; B: romidepsin, lenalidomide, and carfilzomib) were carefully compared in every respect [73]. In the R/R PTCL group, ORRs were similar in two regimens (A: 53%, 8/15 vs. B: 54%, 7/13), but regimen B had a superior CRR than regimen A (A: 13%, 2/15 vs. B: 39%, 5/13) [73]. Compared to regimen A, regimen B showed lower chemotherapeutic toxicity with fewer AEs [73]. However, the AEs of the R/R PTCL group were not acquired. 8) Gemcitabine, as a pyrimidine anti-metabolite, demonstrated single-agent activity in R/R PTCL with an ORR of up to 51% (20/39) and a CRR of 23% (9/39) [75, 76]. However, in a phase II study (NCT01822886), the GEMRO regimen (romidepsin plus gemcitabine) did not show additional advantages on efficacy or safety over single agent romidepsin with a 30% (6/20) ORR and a 15% (3/20) CRR [75]. Another combined regimen of romidepsin plus GDP (gemcitabine, dexamethasone, and cisplatin) was explored in R/R PTCL and DLBCL in a phase I trial (NCT01846390) [77]. Among the PTCL patients, the ORR, 1-year PFS, 1-year OS, mPFS, and mOS were 60% (6/10), 30%, 52%, 5.45 months, and 15.08 months, respectively [77]. The AEs occurring in PTCL were not acquired with a further illustration. 9) Anthracycline antibiotics doxorubicin and romidepsin were reported synergistic in growth inhibition and apoptosis induction in both CTCL cell lines and patient-derived primary CTCL cells [78]. Compared to doxorubicin, liposomal doxorubicin (LD) shows lower cardiotoxicity through reduced myocardial drug accumulation and induction of IFN-related DNA damage resistance [79]. In a phase I trial (NCT01902225), the combination of romidepsin and LD was evaluated in patients with R/R CTCL or PTCL [78]. This regimen demonstrated superior efficacy in CTCL than PTCL in terms of ORR (70% vs. 27%), mPFS (6.9 months vs. 2.1 months), mOS (not reached vs. 17.5 months), the median time to response (mTTR) (2 months vs. 3.5 months), and mDOR (5.1 months vs. 4.2 months) [78]. Moreover, grade 3/4 hematologic AEs occurred more frequently in the PTCL cohort [78]. Markedly, there were no cardiac-related AEs despite high anthracycline exposure [78].

### Belinostat

As a broad-spectrum HDACi and a second-generation analog of vorinostat, belinostat (PXD101) shows antiangiogenic properties and preferential cytotoxicity toward tumor cells, resulting in the apoptosis and cell cycle arrest of some transformed cells [80, 81]. Belinostat was approved by the FDA for the single-agent treatment of R/R PTCL in 2014, based on a phase II single-arm BELIEF trial (NCT00865969) including 120 evaluable cases with R/R PTCL [82–84]. Monotherapy with belinostat yielded an ORR of approximately 25%, also reported in another early phase II trial (NCT00274651) with a CRR of 8.3% (2/24) in the R/R PTCL cohort [83, 85]. In the former, severe AEs occurred in 47.3% (61/129) of patients, recording a higher frequency in nonhematologic AEs [83]. However, one case that died from toxic liver failure without complications was attributed to belinostat [83]. The rare hepatotoxicity of belinostat was presumed to be a result of the sulfonamide-like hepatic reaction due to its chemical properties of sulfonamide hydroxyamide [86].

The exploration of combinatorial therapy for belinostat is still underway. In a phase I trial for newly diagnosed PTCLs (NCT01839097), the addition of belinostat to CHOP (Bel-CHOP) achieved 86% (6/7, 12/14) ORRs in both cohorts with different doses of belinostat and the same dose of CHOP [87]. Of the cases, 43% experienced severe AEs, with febrile neutropenia (17%) occurring most [87]. Synergistic effects between belinostat and proteasome inhibitor ixazomib [88] or dihydrofolate reductase inhibitor pralatrexate [89] have been demonstrated in T-cell lymphoma (TCL) in vitro, providing clues for combination drug trials. In addition, a scale liposomal subcutaneous delivery system of belinostat has been successfully designed for PTCLs to prolong the short half-life of 1.1 h and improve the burst effect and pharmacokinetic properties [90]. However, this novel system temporarily lacks test data in the human body.

### Chidamide

Chidamide (Tucidinostat), a novel selective inhibitor targeting HDAC1, 2, 3, and 10, has demonstrated broad-spectrum antitumor activity. Previous research suggests that chidamide causes tumor cell growth arrest and apoptosis, promotes cellular antitumor immunity, and reverses epithelial–mesenchymal transitions and drug resistance [91]. It acquired approval from the China Food and Drug Administration in 2014 for the treatment of R/R PTCL, based on a multicenter, pivotal phase II trial for R/R PTCL, in which a 28% (22/79) ORR and a 14% (11/79) CR/CRu rate were reported [91]. Angioimmunoblastic T-cell lymphoma (AITL) cases appeared to be more susceptive, with a higher ORR (50%, 5/10) and a CR/CRu rate (40%, 4/10) [91]. For adult T-cell leukemia/lymphoma (ATLL), a 30.4% (7/23) ORR, a 4.3% (1/23) CRR, and an mPFS of 1.7 months were achieved in a phase IIb trial (NCT02955589) of single-agent chidamide [92].

Chidamide-based combined regimens for untreated PTCLs have been studied in recent years. A phase I trial (NCT02809573) and a phase II trial (NCT02987244) evaluated the addition of chidamide to CHOP and CHOEP (CHOP with etoposide) regimens in untreated PTCLs, respectively, with ORRs of 89.3% and 60.2% and CRRs of 57.1% and 40.7% [93, 94]. However, in research on the Chi-CHOEP regimen, patients with AITL showed significantly inferior outcomes with an mPFS of 9.6 months, compared to anaplastic lymphoma kinase-negative anaplastic large cell lymphoma (ALK^−^ALCL) (26.0 months) and PTCL-NOS (19.4 months) [94]. Leukopenia and neutropenia were the most common grade 3/4 hematological AEs in both trials [93, 94]. Another chemotherapy regimen, the CPET regimen (chidamide with prednisone, etoposide, and thalidomide), was evaluated among AITL patients in a multicenter phase II trial (NCT03273452). The ORR and CRR of 51 evaluable cases were 90.2% and 54.9%, respectively [40]. The most common grade 3/4 AE was neutropenia (32.3%), while the other AEs were mainly mild and reversible [40].

### Panobinostat

Panobinostat (LBH-589), a potent oral pan-HDACi, has been reported to have a highly synergistic effect with proteasome inhibitor bortezomib. Their synergy is seemingly attributed to the concurrent inhibition of proteasome and HDAC6, contributing to the accumulation of polyubiquitinated proteins and increased cell stress and apoptosis. In an open-label, multicenter phase II trial (NCT00901147), this combination regimen was evaluated in 25 cases with R/R PTCL, with the majority subtypes being PTCL-NOS (9, 36%) and AITL (8, 32%) [95]. A 43.5% (10/23) ORR and a 21.7% (5/23) CRR were recorded in assessable patients, while two cases discontinued treatment due to peripheral neuropathy, diarrhea, and acute coronary syndrome [95].

### Vorinostat

Vorinostat (SAHA) is an oral pan-HDACi approved for the treatment of R/R CTCL by the FDA in 2006 [82]. In vitro, vorinostat triggers growth arrest and caspase-dependent apoptotic and caspase-independent autophagic cell death [96]. A phase I trial (NCT00787527) evaluated the safety and efficacy of vorinostat combined with six cycles of CHOP in newly diagnosed PTCLs. In addition to 2 cases with premature treatment termination, the remaining 12 all achieved complete remission, whereas 4 experienced disease recurrence [97]. The most common toxicity related to vorinostat was estimated to be diarrhea [97]. In another phase I/II trial (NCT00972842), the regimen of vorinostat in combination with lenalidomide and dexamethasone did not demonstrate satisfactory results, with one complete remission and one partial remission [98]. The regimen of vorinostat plus alisertib was studied in lymphoid malignancies in a phase I trial (NCT01567709) [99]. An 11.8% (4/34) ORR and two CRs only with DLBCL were reported among all cohorts with different dose levels [99]. Two cases of PTCL were enrolled but achieved no response [99]. More vorinostat-based combination therapies for PTCLs are temporarily absent.

### Other potential HDACis for PTCL

**Fig. 3:**
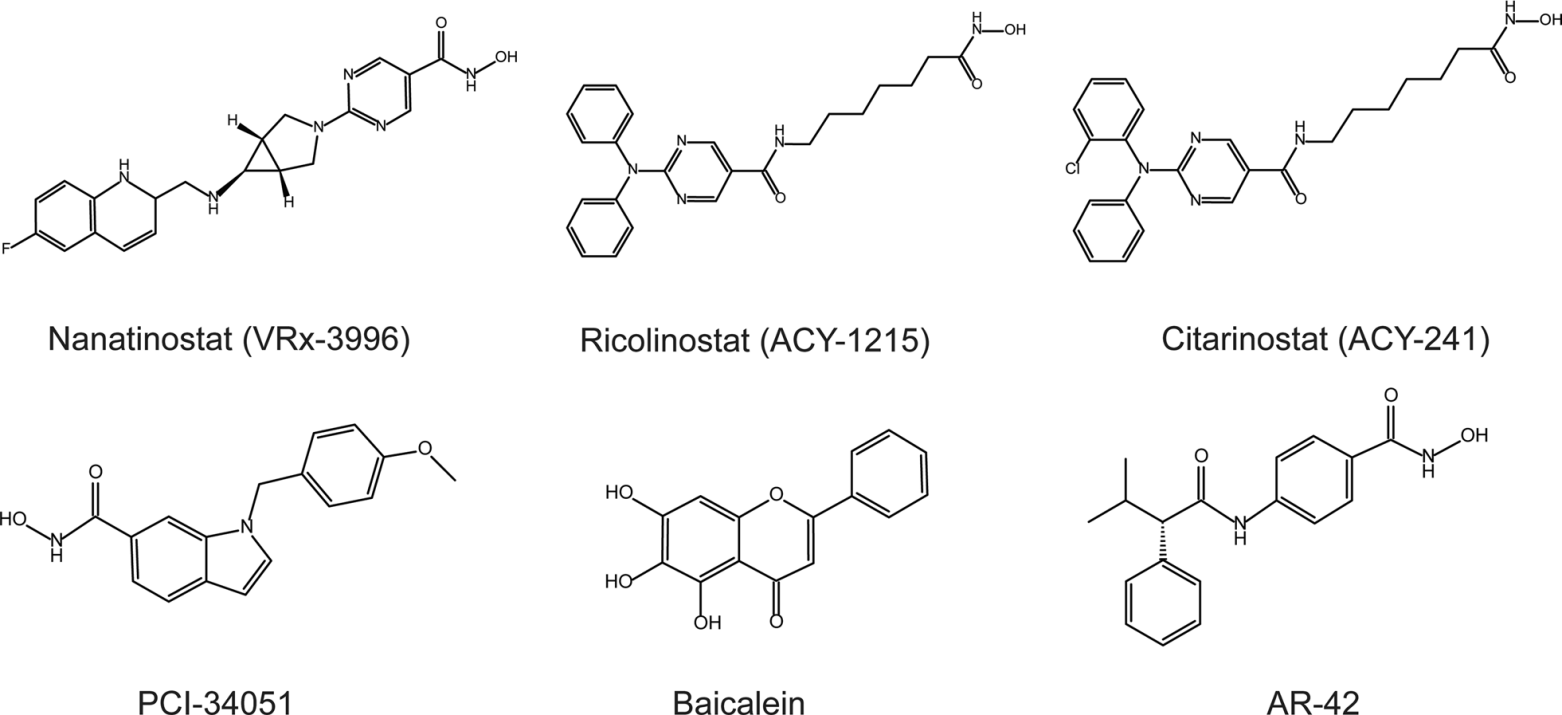
6 potential non-approved HDACis for PTCL. These potential HDACis remaining non-approved and not acquiring comprehensive clinical evaluation in PTCL

Generally, the development of novel agent appeared to be a long-term and intricate procedure. There are many potential HDACis remaining non-approved and not acquiring comprehensive clinical evaluation in PTCL, as shown in Table 3 and Fig. 3.

**Table 3:** Other potential non-approved HDACis for PTCL

Agent	Last update year	Target	Main diseases under research	Highest phase	Clinical trial registration number (e.g.)
Nanatinostat (VRx-3996) [52]	2021	Class I HDACs	EBV^+^ lymphoma	II	NCT03397706, NCT05011058
Ricolinostat (ACY-1215) [100]	2021	HDAC6	R/R lymphoma, MM, and breast cancer	Ib/II	NCT02091063
Citarinostat (ACY-241) [102]	2023	HDAC1, 2, 6	Lymphoma, MM, pancreatic cancer, and NSCLC	I	NCT02400242, NCT02635061
PCI-34051 [103–105]	2021	HDAC8	TCL, CC, HCC, and neuroblastoma	NA	NA
Baicalein [38]	2019	HDAC1, 8	TCL, NSCLC, CC, renal interstitial fibrosis, and influenza	II	NCT03830684, CTR20182427
AR-42 [107]	2018	Pan-HDACs	MM, TCL, BCL, and AML	I	NCT01129193

*NA* Not acquired, *MM* Multiple myeloma, *HL* Hodgkin lymphoma, *TCL* T-cell lymphoma, *BCL* B-cell lymphoma, *FL* Follicular lymphoma, *MSS CRC* microsatellite-stable colorectal cancer, *NHL* Non-Hodgkin lymphoma, *BCP-ALL* B-cell precursor acute lymphoblastic leukemia, *PV* Polycytemia vera, *CMN* Chronic myeloproliferative neoplasms, *DMD* Duchenne muscular dystrophy, *EBV+* Epstein-Barr virus-positive, *R/R* relapsed and refractory, *NSCLC* Non-small cell lung cancer, *CC* Colon cancer, *HCC* Hepatocellular carcinoma, *AML* Acute myeloid leukemia.

Nanatinostat (VRx-3996), selectively targeting Class-I HDACs, can induce cell apoptosis and inhibition of viral and cellular DNA synthesis in EBV^+^ tumor cells, via upregulating the lytic BGLF4 EBV protein kinase and activating ganciclovir [52]. In the phase Ib/II VT3996-201 study (NCT03397706) with 55 patients enrolled (PTCL-NOS, *n* = 5; AITL, *n* = 6), Nanatinostat in combined with valganciclovir (VGCV) achieved a 40% (17/43) ORR and a 19% (8/43) CRR in 43 evaluable patients with histologically confirmed EBV^+^ lymphomas [52]. The most common grade 3/4 AEs across all histologic types included neutropenia (27%), thrombocytopenia (20%), anemia (20%), and lymphopenia (14%) [52].

Ricolinostat (ACY-1215), an oral selective HDAC6 inhibitor with a weak potency against HDAC1 and 2, can disrupt protein homeostasis via the unfolded protein response to induce programmed cell death [100]. In a single-agent phase Ib/II study of ricolinostat (NCT02091063), no complete or partial response was recorded in 16 evaluable R/R lymphoma patients enrolled, despite no dose-limiting toxicities being observed [100]. A combined regimen dually targeting protein degradation pathways, ricolinostat plus proteasome inhibitor bortezomib, was reported profound synergism in both cell-based and in vivo studies [101]. Citarinostat (ACY-241) is a second-generation selective HDAC6 inhibitor, yet with greater inhibition of Class I HDACs due to higher serum concentrations and a form of tablet formulation [100]. Citarinostat plus momelotinib, a JAK1/2 inhibitor, was reported anticancer effects in vitro in hematological malignant cell lines (including FL, MCL, CTCL, ALCL, HL, MM, and CLL) [102]. Multiple cell death mechanisms were recorded, such as activating mitochondrial apoptosis pathways, elevating ROS production, facilitating ER stress, and modulating cell cycle perturbation [102]. Most cell lines showed well susceptibilities to this combined regimen excluding two specific cell lines (Granta-519 and L-1236) [102].

Selective HDAC8 inhibitor PCI-34051 induces caspase-dependent apoptosis in T-cell lymphomas or leukemias cell lines [103]. Deficiency in phospholipase C-γ1 (PLCγ1) rather than in TCR signaling may impair the sensitivity to PCI-34051 [103]. In addition, the tumor-suppressive effect of PCI-34051 was reported as T-cell-dependent and calcium-induced, increasing tumor-infiltrating CD8 T-cells in a preclinical model of hepatocellular carcinoma (HCC) [104]. CD8 T-cell depletion, regulatory T-cell adoptive transfer, Ca^2+^ chelators may cause PCI-34051 to fail in antitumor effect [104]. Without any obvious indications of toxicity, PCI-34051 enhanced the removal of existing hepatomas by anti-PD-L1 therapy [104]. Besides, the synergistic effect of ALK inhibitor and PCI-34051 was reported both in vitro and in vivo in neuroblastoma via blocking the activation of growth receptor survival signaling and shifting the cell cycle arrest [105]. Despite multiple preclinical tests, no clinical trial of PCI-34051 in any malignancy has been registered.

Natural HDAC1 and 8 inhibitor baicalein has been studied in multiple diseases, mainly exhibits antitumor, anti-inflammatory, and anti-fibrotic activities [38]. Baicalein was observed to induce a dose dependent cell death in TCL in vitro via inhibition of thioredoxin system [106]. In another preclinical test in CTCL cell lines, baicalein significantly induced cell apoptosis with a p53 wild type via dramatically raising the level of higher level of acetylation of p53 and proteasome-dependent degradation of HDAC1 [38]. Baicalein appeared superior to traditional HDACis in CTCL due to no influence on the expression of ATP-binding cassette transporter genes [38]. However, existing clinical trials of baicalein have been limited to study its role in influenza.

AR-42, as a pan-HDACi targeting Class I and IIb HDAC, shows antitumor activity in in vitro and in vivo numerous models of solid tumors and hematologic malignancies [107]. In multiple preclinical lymphoma models, AR-42 exhibits a more potent activity than vorinostat [107]. In a phase I trial (NCT01129193) of AR-42, the best response in MM was the minimal response in 17.6% (3/17) patients, while In patients with relapsed lymphoma was a stable disease [107]. Only one PTCL patient that was diagnosed as EBV^+^ AITL was enrolled and achieved progressive disease [107].

## Discussion and future perspectives

HDACs are one of the keys to epigenetic regulation involved in tumorigenesis and tumor maintenance, being promising targets for antitumor agent discovery and development. HDACis have been confirmed to be targeted and available for various tumors, including PTCLs, to improve the poor therapeutic outcomes of the historical standard frontline approach, such as CHOP or CHOP-like regimens. Accumulating evidence suggests that AITL and other T follicular cell-originated PTCL subtypes characteristic of epigenetic disruption have a unique vulnerability to epigenetic inhibitors, including HDACis. However, no additional benefit has been observed by adding chidamide to the CHOEP regimen in untreated AITL, though the high single-agent activity of chidamide has been validated in R/R AITL (50% ORR and 40% CRR) [91, 94]. Hence, it is considered that AITL cases might not significantly profit from intensive chemotherapy.

With the advanced application of next-generation sequencing (NGS), the correlation between mutation profiles, especially in epigenetic genes, and targeted drug therapeutic effects deserves much attention. In a multicenter phase II study (NCT01998035) of R/R PTCL, mutations of genes involved in DNA methylation, histone methylation, or histone acetylation were found more frequently in patients responding to 5-azacytidine plus romidepsin [64]. Clinical responses were more seen in TET2 gene-mutated patients (*n* = 16; ORR 69%; CRR 53%) compared to wild-type mutations (*n *= 5; ORR 40%; CRR 20%), with no statistically significant differences due to small sample size [64]. Whereas, mutation analysis based on NGS may serve as a promising and sensitive biomarker for predicting response and estimating the vulnerability to targeted therapy in patients with PTCL.

Despite the approval of several HDACis for specific cancer treatments, the outcomes of HDACi monotherapies in PTCLs are unsatisfactory and sporadically drug-resistant. Thus, combination therapies of HDACi are still being actively explored to overcome drug resistance and compensatory pathways caused by single target as well as to minimize the side effects. The addition of different kinds of HDACis to CHOP or CHOP-like regimens is not completely extra-beneficial for PTCLs or specific subtypes, as mentioned above. HDACi-based regimens plus other novel targeted agents, such as pembrolizumab [62], 5-azacitidine [64], bendamustine [67], duvelisib [69], tenalisib [70], pralatrexate [71], lenalidomide [73], and carfilzomib [73], hold promising futures for more effective and safer therapeutic outcomes. Combining HDACis with radiotherapy and phototherapy provides new strategies for antitumor therapies due to the inhibition of DNA repair with HDACis [108].

**Fig. 4:**
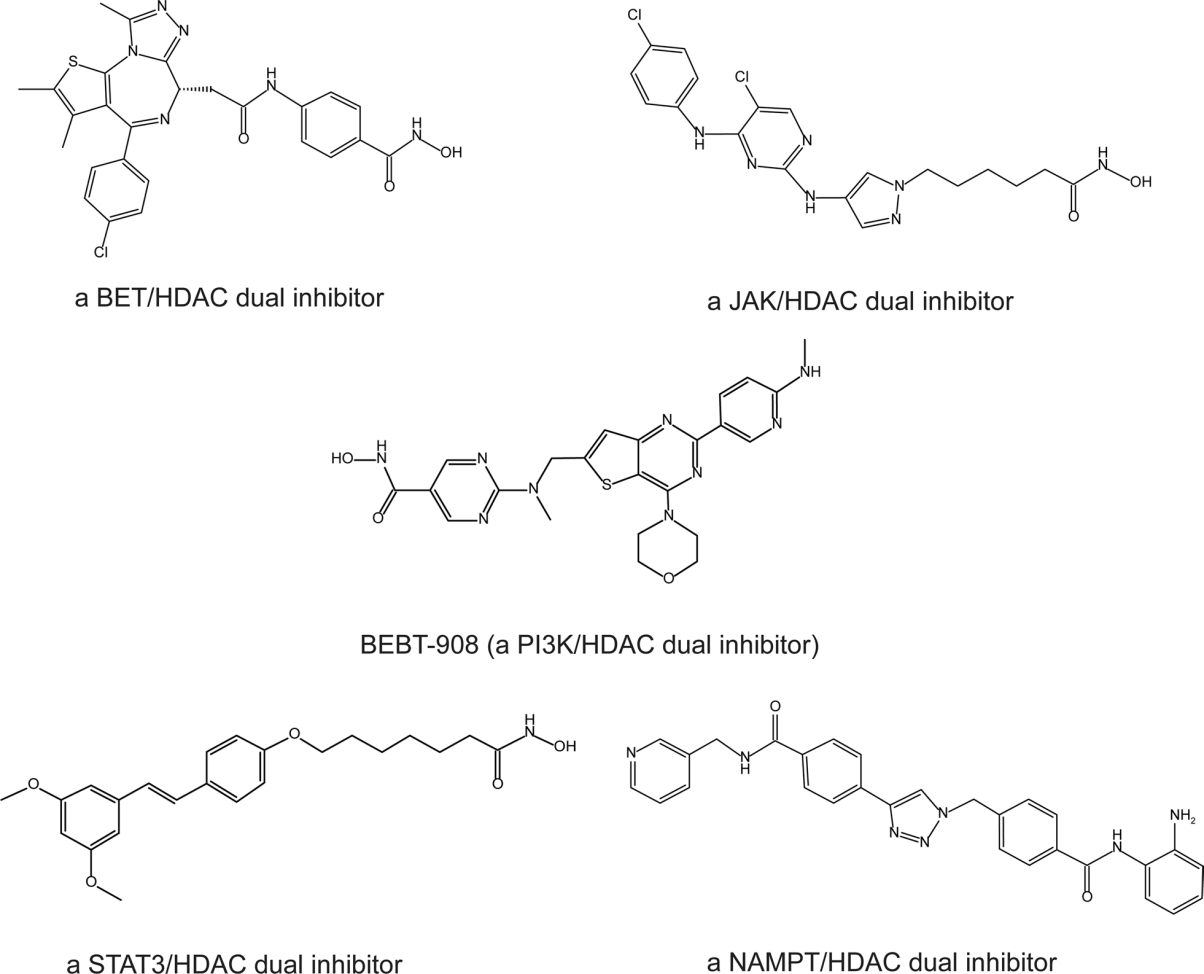
Five kinds of dual HDAC inhibitors in preclinical tests or early clinical trials. Dual HDAC inhibitors may offer more excellent therapeutic benefits in overcoming drug resistance and amplifying synergistic effects. However, the therapeutic potential of novel dual HDACis is urgently warranted further confirmation in clinical trials

Dual inhibitors, which simultaneously aim at two or more targets, may offer more excellent therapeutic benefits over single-acting agents in overcoming drug resistance and amplifying synergistic effects. The discovery and development of HDACi-based multitarget antitumor agents have achieved initial success. Several dual HDAC inhibitors have been accomplished in preclinical tests or early clinical trials (as shown in Fig. 4), such as bromodomain and extra-terminal (BET)/HDAC inhibitors [109], various kinase/HDAC inhibitors [110, 111], STAT3/HDAC inhibitors [112], and Nicotinamide Phosphoribosyltransferase (NAMPT)/HDAC inhibitors [113]. For instance, a novel Janus Kinase (JAK)/HDAC dual inhibitor targeting both JAK2 and HDAC6 has exhibited improved antiproliferative and proapoptotic activities over vorinostat and ruxolitinib in several hematological cell lines [111]. Moreover, a dual PI3K/HDAC inhibitor BEBT-908 can promote a proinflammatory tumor microenvironment and induce immunogenic ferroptosis to synergize with immunotherapy [114]. Of note, BEBT-908 has been further studied in virous clinical trials in China, such as R/R PTCL (Phase II, CTR20210170), R/R DLBCL (Phase II, CTR20200035), advanced non-small cell lung cancer (Phase Ib/II, CTR20213331), and advanced recurrent or metastatic HR^+^/HER2^−^ breast cancer (Phase Ib/II, CTR20213267). More related details can be found on www.chinadrugtrials.org.cn. In general, the therapeutic potential of novel dual HDACis urgently needs to be further confirmed in clinical trials.

Apart from restricted combination regimens and underway multitargeted development, the potential clinical benefits of HDACis are also limited by the insufficient physicochemical properties, selectivity, and potency, which results in inconstant off-target effects and undesirable side effects. HDACi-based prodrugs, an inactive form needing biochemical transformations, are considered an optimistic way to improve medication performance. HDACi-based prodrug strategies are often utilized to enhance targeted location accessibility of drug delivery and overcome deficiencies in the physicochemical properties, such as stability, aqueous solubility, lipophilicity, and oral bioavailability [115]. Of note, romidepsin has prodrug properties and favorable pharmacokinetic properties, making it inactive and stable in blood circulation but active after uptake into tumor cells and resulting in intracellular reduction [115]. Despite plenty of novel HDACi-based prodrugs having been developed, most of the reported ones still need to be thoroughly investigated in terms of in vivo characteristics.

## Conclusions

HDACis have been extensively studied in the treatment of PTCLs, used either alone or in combination. The currently approved HDACis usually demonstrate moderate single-agent activity and controllable incidence of hematological AEs. However, more effective and safe agents or combination regimens are urgently needed to improve the poor prognosis of PTCL patients. Mutation analysis based on NGS may help to predict the efficacy of targeted therapy in PTCL. The development of multitargeted HDACis and HDACi-based prodrugs is a promising strategy, but their efficacy and safety deserve more clinical data to allow verification.
